# Supplementary material for: Improving sexually transmitted infection screening, testing, and treatment among people with HIV: A mixed method needs assessment to inform a multi-site, multi-level intervention and evaluation plan
Source: PLoS One. 2021 Dec 28;16(12):e0261824. doi: 10.1371/journal.pone.0261824 (PMC8714108; doi:10.1371/journal.pone.0261824)
Supplement: S1 File — (PDF) [file pone.0261824.s001.pdf]

Clinical Team Member Interview Questions

<u>Administration Notes</u>: These survey questions will be asked onsite and audiotaped during in person-qualitative interviews during clinical demonstration site visits by the Rutgers University team in a private room provided by the site. This survey will be administered to 3 care team members (the Change Champion, a clinical prescriber [e.g., MD, DO, NP, PA] and a clinical non-prescriber [e.g., RN, SW, MA] at each of the 9 clinical demonstration sites. Audiotapes of responses will be transcribed using NVivo V12.0 for qualitative analysis.

<u>Instructions</u>: As each question is asked, please provide a response that most reflects your perspective as a member of the clinic team. Please allow for an estimated interview time of up to 60 minutes.

Gonorrhea and Chlamydia Policy and Procedure

1. What are your clinic's current policy and procedure related to gonorrhea and chlamydia screening?
2. What are your clinic's current policy and procedure related to gonorrhea and chlamydia testing?
3. What are your clinic's current policy and procedure for gonorrhea and chlamydia treatment?
4. What are your clinic's current policy and procedure for follow-up regarding gonorrhea and chlamydia infection(s)?
5. Which of these policies and procedure are followed as recommended?

[Obtain a copy of all policies and procedures]

Gonorrhea and Chlamydia Screening and Testing Barriers and Challenges

6. What barriers and challenges do you or your clinical team members encounter when screening for gonorrhea and chlamydia from each of the following 3 anatomical sites?
7. What barriers and challenges do you or your clinical team members encounter when testing for gonorrhea and chlamydia from each of the following 3 anatomical sites?
    - Genital/urine:
    - Oropharyngeal/throat:
    - Rectal:

Supports for Gonorrhea Screening, Testing, Treatment, and Follow-Up

8. If you have any recommendations for making <u>gonorrhea screening</u> more routine or easier for providers and patients at this clinic, what are those recommendations?
9. If you have any recommendations for making <u>gonorrhea testing</u> more routine or easier for providers and patients at this clinic, what are those recommendations?
10. If you have any recommendations for making <u>gonorrhea treatment</u> more routine or easier for providers and patients at this clinic, what are those recommendations?
11. If you have any recommendations for making <u>gonorrhea follow-up</u> more routine or easier for providers and patients at this clinic, what are those recommendations?

Supports for Chlamydia Screening, Testing, Treatment, and Follow-Up

12. If you have any recommendations for making <u>chlamydia screening</u> more routine or easier for providers and patients at this clinic, what are those recommendations?
13. If you have any recommendations for making <u>chlamydia testing</u> more routine or easier for providers and patients at this clinic, what are those recommendations?

14. If you have any recommendations for making chlamydia treatment more routine or easier for providers and patients at this clinic, what are those recommendations?
15. If you have any recommendations for making chlamydia follow-up more routine or easier for providers and patients at this clinic, what are those recommendations?

Syphilis Policy and Procedure

16. What are your clinic's current policy and procedure related to syphilis screening?
17. What are your clinic's current policy and procedure related to syphilis testing?
18. What are your clinic's current policy and procedure for syphilis treatment?
19. What are your clinic's current policy and procedure for follow-up regarding syphilis infection(s)?
20. Which of these policies and procedure are followed as recommended?

Syphilis Screening and Testing Barriers and Challenges

21. What barriers and challenges do you or your clinical team members encounter when screening for syphilis?
22. What barriers and challenges do you or your clinical team members encounter when testing for syphilis?

Supports for Syphilis Screening, Testing, Treatment, and Follow-Up

23. If you have any recommendations for making syphilis screening more routine or easier for providers and patients at this clinic, what are those recommendations?
24. If you have any recommendations for making syphilis testing more routine or easier for providers and patients at this clinic, what are those recommendations?
25. If you have any recommendations for making syphilis treatment more routine or easier for providers and patients at this clinic, what are those recommendations?
26. If you have any recommendations for making syphilis follow-up more routine or easier for providers and patients at this clinic, what are those recommendations?

HIV Screening and Testing Barriers and Challenges

27. What barriers and challenges do you or your clinical team members encounter when screening for HIV among those (HIV-uninfected or unknown status) at-risk for HIV?
28. What barriers and challenges do you or your clinical team members encounter when testing for HIV among those (HIV-uninfected or unknown status) at-risk for HIV?

Cultural Competence

29. Describe ways your clinical team members have been involved in increasing cultural competence to caring for diverse populations of people living with HIV (ethnicity, age, sexual orientation, gender identity, mental illness, etc.).
30. Describe ways your clinical team members have been involved in increasing cultural competence to caring for diverse populations of those (HIV-uninfected or unknown status) at-risk for HIV (ethnicity, age, sexual orientation, gender identity, mental illness, etc.).
