# Supplementary material for: Improving sexually transmitted infection screening, testing, and treatment among people with HIV: A mixed method needs assessment to inform a multi-site, multi-level intervention and evaluation plan
Source: PLoS One. 2021 Dec 28;16(12):e0261824. doi: 10.1371/journal.pone.0261824 (PMC8714108; doi:10.1371/journal.pone.0261824)
Supplement: S2 File — (PDF) [file pone.0261824.s002.pdf]

STI SPNS Pre-Intervention Data Survey: 2016-2017

<u>Administration Notes</u>: This survey will be administered online via REDCap (Research Electronic Data Capture), an online survey platform, to the designated Change Champion at each of the 9 clinical demonstration sites prior to an in-person site visits by Rutgers University investigators.

Instructions: Please answer each question below as accurately as possible. Please allow for an estimated completion time of up to 60 minutes. Aggregate data results for the calendar years 2016 & 2017 will be utilized in the intervention selection process for your Ryan White HIV/AIDS Program (RWHAP)-funded HIV primary care clinic.

<u>FOR CALENDAR YEARS (JANUARY 1 – DECEMBER 31) 2016 & 2017:</u>

1) Total number of people living with HIV (<u>**PLWH)**</u> by calendar year seen at your RWHAP– funded primary care clinic
   2016: __________
   2017: __________
2) Total number of individuals (HIV-uninfected or unknown HIV status) <u>**at-risk for HIV infection**</u> by calendar year seen at your RWHAP– funded primary care clinic
   2016: __________
   2017: __________
3) Of the total number of individual <u>PLWH</u> seen in your RWHAP-funded primary care clinic over the calendar year:

	2016	2017
<u>Number tested</u> for **genital or urine chlamydia/ gonorrhea**	Not tested_____<br>Once/year_____<br>2-3 times/year____<br>4-5 times/year____<br>>5 times/year____	Not tested____<br>Once/year____<br>2-3 times/year____<br>4-5 times/year____<br>>5 times/year____
<u>Number tested</u> for **oropharyngeal chlamydia/ gonorrhea**	Not tested______<br>Once/year_____<br>2-3 times/year_____<br>4-5 times/year_____<br>>5 times/year_____	Not tested______<br>Once/year_____<br>2-3 times/year_____<br>4-5 times/year_____<br>>5 times/year_____
<u>Number tested</u> for **rectal chlamydia/ gonorrhea**	Not tested_______<br>Once/year______<br>2-3 times/year_____<br>4-5 times/year_____<br>>5 times/year______	Not tested_______<br>Once/year______<br>2-3 times/year_____<br>4-5 times/year_____<br>>5 times/year______
<u>Number tested</u> for **syphilis**	Not tested_______<br>Once/year_____<br>2-3 times/year_____<br>4-5 times/year_____<br>>5 times/year_____	Not tested______<br>Once/year_____<br>2-3 times/year____<br>4-5 times/year_____<br>>5 times/year_____

4) Total number of individual <u>PLWH</u> diagnosed with a positive chlamydia urine or genital test result in the calendar year
   2016: __________
   2017: __________
5) Total number of individual <u>PLWH</u> diagnosed with a positive gonorrhea urine or genital test result in the calendar year
   2016: __________

2017: __________

6) Total number of individual <u>PLWH</u> diagnosed with a positive chlamydia oropharyngeal test result in the calendar year
   2016: __________
   2017: __________
7) Total number of individual <u>PLWH</u> diagnosed with a positive gonorrhea oropharyngeal test result in the calendar year
   2016: __________
   2017: __________
8) Total number of individual <u>PLWH</u> diagnosed with a positive chlamydia rectal test result in the calendar year
   2016: __________
   2017: __________
9) Total number of individual <u>PLWH</u> diagnosed with a positive gonorrhea rectal test result in the calendar year
   2016: __________
   2017: __________
10) Total number of individual <u>PLWH</u> diagnosed with an untreated syphilis infection in the calendar year
   2016: __________
   2017: __________
11) A) Total number of individual <u>PLWH</u> diagnosed with recurrent or reinfection with chlamydia or gonorrhea or syphilis in the same calendar year
   2016: __________
   2017: __________

   B) Total number of individual <u>PLWH</u> diagnosed with 2 or more types of STIs (chlamydia, gonorrhea, or syphilis) in the same calendar year
   2016: __________
   2017: __________
12) Approximately what percentage of your PLWH patients diagnosed with an STI in the past year had their partner(s) treated in your clinic? ____________ %
13) Of the total number of individuals (HIV-uninfected or unknown HIV status) <u>at-risk</u> for HIV infection seen in your clinic over the calendar year *(if your RWHAP-funded clinic does not do STI testing on HIV-uninfected or status unknown people, skip questions 13-21)*

	2016	2017
Number tested for **genital or urine chlamydia/ gonorrhea**	Not tested______<br>Once/year_____<br>2-3 times/year____<br>4-5 times/year____<br>>5 times/year____	Not tested_____<br>Once/year_____<br>2-3 times/year____<br>4-5 times/year____<br>>5 times/year_____
Number tested for **oropharyngeal chlamydia/ gonorrhea**	Not tested______<br>Once/year____<br>2-3 times/year____<br>4-5 times/year____<br>>5 times/year___	Not tested______<br>Once/year____<br>2-3 times/year____<br>4-5 times/year____<br>>5 times/year____
Number tested for **rectal chlamydia/ gonorrhea**	Not tested_____<br>Once/year____<br>2-3 times/year___<br>4-5 times/year___<br>>5 times/year____	Not tested______<br>Once/year____<br>2-3 times/year____<br>4-5 times/year____<br>>5 times/year____

Number tested for **syphilis**	Not tested____<br>Once/year____<br>2-3 times/year___<br>4-5 times/year___<br>>5 times/year____	Not tested____<br>Once/year____<br>2-3 times/year____<br>4-5 times/year____<br>>5 times/year____

14) Total number of individuals (HIV-uninfected or unknown HIV status) **at-risk** for HIV infection diagnosed with a positive chlamydia urine or genital test result in the calendar year

2016: __________
2017: __________

15) Total number of individuals (HIV-uninfected or unknown HIV status) **at-risk** for HIV infection diagnosed with a positive gonorrhea urine or genital test result in the calendar year

2016: __________
2017: __________

16) Total number of individuals (HIV-uninfected or unknown HIV status) **at-risk** for HIV infection diagnosed with a positive chlamydia oropharyngeal test result in the calendar year

2016: __________
2017: __________

17) Total number of individuals (HIV-uninfected or unknown HIV status) **at-risk** for HIV infection diagnosed with a positive gonorrhea oropharyngeal test result in the calendar year

2016: __________
2017: __________

18) Total number of individuals (HIV-uninfected or unknown HIV status) **at-risk** for HIV infection diagnosed with a positive chlamydia rectal test result in the calendar year

2016: __________
2017: __________

19) Total number of individuals (HIV-uninfected or unknown HIV status) **at-risk** for HIV infection diagnosed with a positive gonorrhea rectal test result in the calendar year

2016: __________
2017: __________

20) Total number of individuals (HIV-uninfected or unknown HIV status) **at-risk** for HIV infection diagnosed with an untreated syphilis infection in the calendar year

2016: __________
2017: __________

21) **A)** Total number of individuals (HIV-uninfected or unknown HIV status) **at-risk** for HIV infection diagnosed with recurrent or reinfection with chlamydia or gonorrhea or syphilis in the calendar year

2016: __________
2017: __________

B) Total number of individuals (HIV-uninfected or unknown HIV status) **at-risk** for HIV infection diagnosed with 2 or more types of STIs (chlamydia, gonorrhea, or syphilis) in the same calendar year

2016: __________
2017: __________

22) Total number of individual **adolescents/young adults** (ages 13-29 years inclusive) **living with HIV** by calendar year seen at clinic

2016: __________
2017: __________

23) Of all **adolescents/young adults** (ages 13-29 years inclusive) **living with HIV** seen in your clinic over the calendar year:

	2016	2017

Number tested for<br>**genital or urine chlamydia/ gonorrhea**	Not tested______<br>Once/year_____<br>2-3 times/year____<br>4-5 times/year____<br>>5 times/year____	Not tested_____<br>Once/year_____<br>2-3 times/year____<br>4-5 times/year____<br>>5 times/year_____
Number tested for<br>**oropharyngeal chlamydia/ gonorrhea**	Not tested______<br>Once/year____<br>2-3 times/year____<br>4-5 times/year____<br>>5 times/year___	Not tested______<br>Once/year____<br>2-3 times/year____<br>4-5 times/year____<br>>5 times/year____
Number tested for<br>**rectal chlamydia/ gonorrhea**	Not tested_____<br>Once/year____<br>2-3 times/year___<br>4-5 times/year___<br>>5 times/year____	Not tested______<br>Once/year____<br>2-3 times/year____<br>4-5 times/year____<br>>5 times/year____
Number tested for<br>**syphilis**	Not tested____<br>Once/year____<br>2-3 times/year___<br>4-5 times/year___<br>>5 times/year____	Not tested____<br>Once/year____<br>2-3 times/year____<br>4-5 times/year____<br>>5 times/year____

24) Total number of individual **adolescents/young adults** **living with HIV** diagnosed with a positive chlamydia urine or genital test result in the calendar year
2016: __________
2017: __________

25) Total number of individual **adolescents/young adults** **living with HIV** diagnosed with a positive gonorrhea urine or genital test result in the calendar year
2016: __________
2017: __________

26) Total number of individual **adolescents/young adults** **living with HIV** diagnosed with a positive chlamydia oropharyngeal test result in the calendar year
2016: __________
2017: __________

27) Total number of individual **adolescents/young adults** living with HIV diagnosed with a positive gonorrhea oropharyngeal test result in the calendar year
2016: __________
2017: __________

28) Total number of individual **adolescents/young adults** **living with HIV** diagnosed with a positive chlamydia rectal test result in the calendar year
2016: __________
2017: __________

29) Total number of individual **adolescents/young adults** **living with HIV** diagnosed with a positive gonorrhea rectal test result in the calendar year
2016: __________
2017: __________

30) Total number of individual **adolescents/young adults** **living with HIV** diagnosed with an untreated syphilis infection in the calendar year

2016: __________
2017: __________

31) A) Total number of individual <u>adolescents/young adults</u> **living with HIV** diagnosed with recurrent or reinfection with chlamydia or gonorrhea or syphilis in the same calendar year
2016: __________
2017: __________
B) Total number of individual <u>adolescents/young adults</u> **living with HIV** diagnosed with 2 or more types of STIs (chlamydia, gonorrhea, or syphilis) in the same calendar year
2016: __________
2017: __________

32) Total number of individual <u>transgender women</u> **living with HIV** by calendar year seen at clinic
2016: __________
2017: __________

33) Of all <u>transgender women</u> **living with HIV** seen in your clinic over the calendar year:

	2016	2017
Number tested for **genital or urine chlamydia/ gonorrhea**	Not tested______<br>Once/year_____<br>2-3 times/year____<br>4-5 times/year____<br>>5 times/year____	Not tested_____<br>Once/year_____<br>2-3 times/year____<br>4-5 times/year____<br>>5 times/year_____
Number tested for **oropharyngeal chlamydia/ gonorrhea**	Not tested______<br>Once/year____<br>2-3 times/year____<br>4-5 times/year____<br>>5 times/year___	Not tested______<br>Once/year____<br>2-3 times/year____<br>4-5 times/year____<br>>5 times/year____
Number tested for **rectal chlamydia/ gonorrhea**	Not tested_____<br>Once/year____<br>2-3 times/year___<br>4-5 times/year___<br>>5 times/year____	Not tested______<br>Once/year____<br>2-3 times/year____<br>4-5 times/year____<br>>5 times/year____
Number tested for **syphilis**	Not tested____<br>Once/year____<br>2-3 times/year___<br>4-5 times/year___<br>>5 times/year____	Not tested____<br>Once/year____<br>2-3 times/year____<br>4-5 times/year____<br>>5 times/year____

34) Total number of individual <u>transgender women</u> **living with HIV** diagnosed with a positive chlamydia urine or genital test result in the calendar year
2016: __________
2017: __________

35) Total number of individual <u>transgender women</u> **living with HIV** diagnosed with a positive gonorrhea urine or genital test result in the calendar year
2016: __________
2017: __________

36) Total number of individual <u>transgender women</u> **living with HIV** diagnosed with a positive chlamydia oropharyngeal test result in the calendar year
2016: __________

2017: __________

37) Total number of individual <u>transgender women</u> **living with HIV** diagnosed with a positive gonorrhea oropharyngeal test result in the calendar year

2016: __________

2017: __________

38) Total number of individual <u>transgender women</u> **living with HIV** diagnosed with a positive chlamydia rectal test result in the calendar year

2016: __________

2017: __________

39) Total number of individual <u>transgender women</u> **living with HIV** diagnosed with a positive gonorrhea rectal test result in the calendar year

2016: __________

2017: __________

40) Total number of individual <u>transgender women</u> **living with HIV** diagnosed with an untreated syphilis infection in the calendar year

2016: __________

2017: __________

41) **A)** Total number of individual <u>transgender women</u> **living with HIV** diagnosed with recurrent or reinfection with chlamydia or gonorrhea or syphilis in the same calendar year

2016: __________

2017: __________

B) Total number of individual <u>transgender women</u> **living with HIV** diagnosed with 2 or more types of STIs (chlamydia, gonorrhea, or syphilis) in the same calendar year

2016: __________

2017: __________

42) Total number of <u>pregnant individuals</u> **living with HIV** by calendar year seen at clinic

2016: __________

2017: __________

43) Of all <u>pregnant individuals</u> **living with HIV** seen at your clinic:

A) Number tested for **genital or urine chlamydia/gonorrhea**

- Not tested ___________________
- In the first trimester _________________
- In the second trimester _______________
- In the third trimester ________________

B) Number tested for **syphilis**

- Not tested _________________
- In the first trimester ________________
- In the second trimester _______________
- In the third trimester ________________

44) Total number of <u>pregnant individuals</u> **living with HIV** diagnosed with a positive chlamydia urine or genital test result in the calendar year

2016: __________

2017: __________

45) Total number of <u>pregnant individuals</u> **living with HIV** diagnosed with a positive gonorrhea urine or genital test result in the calendar year

2016: __________

2017: __________

46) Total number of individual <u>pregnant individuals</u> **living with HIV** diagnosed with an untreated syphilis infection in the calendar year

2016: __________

2017: __________

47) Total number of individual <u>men who have sex with men (MSM)</u> **living with HIV** by calendar year seen at clinic

2016: __________
2017: __________

48) Of all <u>MSM</u> **living with HIV** seen in your clinic over the calendar year:

	2016	2017
Number tested for **genital or urine chlamydia/gonorrhea**	Not tested______<br>Once/year_____<br>2-3 times/year____<br>4-5 times/year____<br>>5 times/year____	Not tested_____<br>Once/year_____<br>2-3 times/year____<br>4-5 times/year____<br>>5 times/year_____
Number tested for **oropharyngeal chlamydia/gonorrhea**	Not tested______<br>Once/year____<br>2-3 times/year____<br>4-5 times/year____<br>>5 times/year___	Not tested______<br>Once/year____<br>2-3 times/year____<br>4-5 times/year____<br>>5 times/year____
Number tested for **rectal chlamydia/gonorrhea**	Not tested_____<br>Once/year____<br>2-3 times/year___<br>4-5 times/year___<br>>5 times/year____	Not tested______<br>Once/year____<br>2-3 times/year____<br>4-5 times/year____<br>>5 times/year____
Number tested for **syphilis**	Not tested____<br>Once/year____<br>2-3 times/year___<br>4-5 times/year___<br>>5 times/year____	Not tested____<br>Once/year____<br>2-3 times/year____<br>4-5 times/year____<br>>5 times/year____

49) Total number of individual <u>MSM</u> **living with HIV** diagnosed with a positive chlamydia urine or genital test result in the calendar year

2016: __________
2017: __________

50) Total number of individual <u>MSM</u> **living with HIV** diagnosed with a positive gonorrhea urine or genital test result in the calendar year

2016: __________
2017: __________

51) Total number of individual <u>MSM</u> **living with HIV** diagnosed with a positive chlamydia oropharyngeal test result in the calendar year

2016: __________
2017: __________

52) Total number of individual <u>MSM</u> **living with HIV** diagnosed with a positive gonorrhea oropharyngeal test result in the calendar year

2016: __________
2017: __________

53) Total number of individual <u>MSM</u> **living with HIV** diagnosed with a positive chlamydia rectal test result in the calendar year

2016: __________
2017: __________

54) Total number of individual <u>MSM</u> **living with HIV** diagnosed with a positive gonorrhea rectal test result in the calendar year

2016: __________

2017: __________

55) Total number of individual <u>MSM</u> **living with HIV** diagnosed with an untreated syphilis infection in the calendar year

2016: __________

2017: __________

56) **A)** Total number of individual <u>MSM</u> **living with HIV** diagnosed with recurrent or reinfection with chlamydia or gonorrhea or syphilis in the same calendar year

2016: __________

2017: __________

B) Total number of individual <u>MSM</u> **living with HIV** diagnosed with 2 or more types of STIs (chlamydia, gonorrhea, or syphilis) in the same calendar year

2016: __________

2017: __________
