# Supplementary material for: Improving sexually transmitted infection screening, testing, and treatment among people with HIV: A mixed method needs assessment to inform a multi-site, multi-level intervention and evaluation plan
Source: PLoS One. 2021 Dec 28;16(12):e0261824. doi: 10.1371/journal.pone.0261824 (PMC8714108; doi:10.1371/journal.pone.0261824)
Supplement: S3 File — (PDF) [file pone.0261824.s003.pdf]

Clinical Team Member Process, Attitudes, & Beliefs Survey

Administration Notes: This survey will be administered online via REDCap (Research Electronic Data Capture), an online survey platform, to the designated Change Champion, a prescribing clinician (i.e., MD, DO, NP, or PA), and a non-prescribing clinical provider (i.e., RN, SW, MA) at each of the 9 clinical demonstration sites prior to an in-person site visits by Rutgers University investigators.

Instructions: Please answer the following questions based upon your current clinical processes in your primary care Ryan White HIV/AIDS Program funded clinic. Please allow for an estimated completion time of up to 60 minutes.

1. All patients have a comprehensive sexual history taken on **intake** to the clinic which includes at least: number of different male partners, number of different female partners, types of intercourse (oral-genital, oral-anal, vaginal, anal receptive and insertive), history of sexually transmitted infections (STIs), contraception history and desires, history of forced/abusive sex, history of sex for trade/exchange, history of finding sexual partners online.

- true
- false
- we do a sexual history, but not as comprehensive (If this option is selected, then ask, what questions are included in your initial sexual history: _________________)
- we do a sexual history, but each provider does it differently and documents in a text box.

2. Follow-up sexual histories are taken: (select all that apply)

- at the “annual” visit (once/year)
- at semi-annual visits
- at quarterly visits
- at acute care visits when symptomatic for an STI

3. Follow-up sexual histories are done with: (select all that apply)

- all patients 13 years old and older
- all patients 18 years old and older
- all visits of adolescents and young adults (ages 13-29 years)
- all sexually active adult men who have sex with men

- all sexually active adult women
- all sexually active transgender patients
- all sexually active men who have sex with women

4. Sexual history taking is done by which means: (select all that apply)

- patient completed paper survey prior to coming to clinic
- patient completed paper survey in clinic prior to being seen by provider(s)
- prescribing provider asks patient questions
- non-prescribing provider asks patient questions
- patient completed online survey before coming to clinic
- patient completed online survey in clinic prior to being seen by provider(s), such as computer-assisted self-interview or CASI

5. For adolescent and adult women living with HIV, which tests are done at intake based on sexual history? (select all that apply)

- syphilis RPR/FTA (in either order)
- syphilis IgG
- vaginal gonorrhea/chlamydia (GC/CT) nucleic acid amplification test (NAAT)
- rectal GC/CT NAAT
- oropharyngeal GC/CT NAAT
- urine GC/CT NAAT
- GC culture(s)
- rapid GC/CT test
- rapid syphilis test
- none of these tests are done

6. For adolescent and adult men living with HIV, which tests are done at intake based on sexual history? (select all that apply)

- syphilis RPR/FTA (in either order)
- syphilis IgG
- penis GC/CT NAAT
- rectal GC/CT NAAT
- oropharyngeal GC/CT NAAT
- urine GC/CT NAAT

- GC culture(s)
- rapid GC/CT test
- rapid syphilis test
- none of these tests are done

7. For adolescent and adult transgender women living with HIV, which tests are done at intake based on sexual history? (select all that apply)

- syphilis RPR/FTA (in either order)
- syphilis IgG
- genital GC/CT NAAT
- rectal GC/CT NAAT
- oropharyngeal GC/CT NAAT
- urine GC/CT NAAT
- GC culture(s)
- rapid GC/CT test
- rapid syphilis test
- none of these tests are done

8. For adolescent and adult transgender men living with HIV, which tests are done at intake based on sexual history? (select all that apply)

- syphilis RPR/FTA (in either order)
- syphilis IgG
- genital GC/CT NAAT
- rectal GC/CT NAAT
- oropharyngeal GC/CT NAAT
- urine GC/CT NAAT
- GC culture(s)
- rapid GC/CT test
- rapid syphilis test
- none of these tests are done

9. For <u>sexually active</u> adolescents and adults living with HIV, how often do you test for STIs (syphilis, gonorrhea, chlamydia) after intake? (select all that apply)

- annually (once/year)
- if symptomatic
- every 6 months for ____________________ (specify population, e.g., MSM, adolescents/young adults)
- every 3-4 months for ___________________ (specify population, e.g., MSM, adolescents/young adults)
- during pregnancy once
- during first and third trimester of pregnancy
- our clinic does not test for STIs, we refer to another provider or Department of Health (DOH) Sexually Transmitted Disease (STD) clinic

  If answers "our clinic does not test for STIs", for what reason(s)? (select all that apply)
  - our RWHAP clinic only provides HIV-specific care
  - we are part of a bigger clinic, and send patients for STI-related care to another provider within our health center/clinic
  - it is too time consuming, and HIV expert provider time is limited in our clinic
  - provider/staff discomfort with doing routine sexual history taking and STI testing
  - other, please specify: ____

10. For HIV-uninfected but at risk for infection adolescents and adults, which tests are done at intake based on sexual history? (select all that apply)

- syphilis RPR/FTA (in either order)
- syphilis IgG
- penis GC/CT NAAT
- rectal GC/CT NAAT
- oropharyngeal GC/CT NAAT
- urine GC/CT NAAT
- GC culture(s)
- rapid GC/CT test
- rapid syphilis test
- rapid HIV antibody test

- rapid HIV antigen/antibody test
- non-rapid HIV antigen/antibody test
- non-rapid HIV antibody test
- none of these tests are done
- we do not provide STI testing of HIV-uninfected or status unknow people

11. If a patient wants to be seen for a possible STI (testing and/or treatment) and does not have an appointment with your clinic, what would be your clinic's most likely response?

- come into the clinic and we'll make sure you get seen today
- let's make you an appointment to come in to the clinic, but it will not be today
- we're going to have to have you go to the DOH STD clinic or to another provider to be seen
- you should go to an emergency department or urgent care center

12. Who does the NAAT or culture specimen collections for GC and CT? (select all that apply)

- patient self-collects
- prescribing provider collects
- non-prescribing clinical team member (e.g., RN) collects
- not applicable – we do not do GC/CT NAAT or GC cultures in our HIV care clinic

13. Where does the patient get blood drawn?

- on-site in the clinic, we have a phlebotomist
- on-site in the clinic, we don't have a phlebotomist, but one of our clinical staff draws the blood
- an off-site or out-of-clinic laboratory

  If blood is drawn at an off-site or out-of-clinic laboratory, what percentage of patients are successful in getting blood work done within a week?

    - 25% or less
    - 26-50%
    - 51-75%
    - 76-100%

14. Which type of laboratory do you use for each of the following STI tests?

- genital or urine GC/CT:
    - hospital lab

  - state DOH lab
  - commercial lab
  - we use rapid point-of-care tests
  - other, please specify: ______________________
- throat GC/CT:
  - hospital lab
  - state DOH lab
  - commercial lab
  - we use rapid point-of-care tests
  - other, please specify: ______________________
- rectal GC/CT:
  - hospital lab
  - state DOH lab
  - commercial lab
  - we use rapid point-of-care tests
  - other, please specify: ______________________
- syphilis:
  - hospital lab
  - state DOH lab
  - commercial lab
  - we use rapid point-of-care tests
  - other, please specify: ______________________
- HIV Ab or HIV Ag/Ab:
  - hospital lab
  - state DOH lab
  - commercial lab
  - we use rapid point-of-care tests
  - other, please specify: ______________________

15. If the patient has symptoms of an STI at presentation to the clinic (e.g., urethral discharge, proctitis, non-tender, ulcerated genital lesion consistent with primary syphilis, a rash consistent with secondary syphilis), how would you/your clinic address it?

- send non-rapid tests for GC/CT, syphilis but not treat until results are back
- do rapid tests for GC/CT, syphilis and treat on-site based on symptoms and/or positive rapid test results
- do rapid tests for GC/CT and give prescriptions to treat any positive rapid test result
- send non-rapid tests (GC/CT, syphilis) and give prescriptions for meds to be taken off-site or for injectables to be bought and brought back to clinic for injecting (if meds not available in clinic)
- Other, please specify: ___________________________________

16. If an STI test result comes back positive for an asymptomatic patient who are tested, who notifies the patient to come back to the clinic for treatment or to take medication(s) called into the patient's pharmacy?

- a prescribing provider
- a nurse
- a case manager
- a patient navigator
- a social worker
- other, please specify: _____________________

17. On the average, how quickly are patients brought back in to clinic for a positive STI test result after being tested?

- within 1-3 days
- within 4-6 days
- 7-10 days
- more than 10 days
- Patients are not brought back to our clinic; they are referred to an STD clinic or a prescription is called in their pharmacy

18. Which techniques do you use to get patients back to clinic for treatment of a positive chlamydia test result? (select all that apply)

- we order the prescription for delivery or pick-up by patient at a pharmacy, and the patient self-administers
- cell or home phone call
- cell phone text
- DOH Disease Intervention Specialist (DIS)

- patient navigator/community health worker
- use of a hospital/clinic based communication app (e.g., My Chart)
- other, please specify: ______________________________

19. Which techniques do you use to get patients back to clinic for treatment of a positive gonorrhea test result? (select all that apply)

- cell or home phone call
- cell phone text
- DOH DIS
- patient navigator/community health worker
- use of a hospital/clinic based communication app (e.g., My Chart)
- other, please specify: ________________________

20. Which techniques do you use to get patients back to clinic for treatment of a positive syphilis test result? (select all that apply)

- cell or home phone call
- cell phone text
- DOH DIS
- patient navigator/community health worker
- use of a hospital/clinic based communication app (e.g., My Chart)
- other, please specify: _____________________________

21. For partner(s) treatment of chlamydia, which of the following do you do in your clinic? (select all that apply)

- we refer to the DOH STD Clinic
- we provide expedited partner treatment (EPT) through the patient
- we provide the treatment in our clinic if patient brings partner(s) in
- we provide HIV testing to the partner in our clinic if not known to be living with HIV
- we refer patients to the DOH DIS
- depends if the patient is known to be living with HIV – if yes, we see in our RWHAP clinic otherwise we refer out
- other, please specify: _________________________________

22. For partner(s) treatment of gonorrhea, which of the following do you do in your clinic? (select all that apply)

- we refer to the DOH STD Clinic

- we provide expedited partner treatment (EPT) through the patient
- we provide the treatment in our clinic if patient brings partner(s) in
- we provide HIV testing to the partner in our clinic if not known to be living with HIV
- we refer to the DOH DIS depends if the patient is known to be living with HIV – if yes, we see in our RWHAP clinic otherwise we refer out
- other, please specify: ____

23. For partner(s) treatment of syphilis, which of the following do you do in your clinic? (select all that apply)

- we refer to the DOH STD Clinic
- we provide the treatment in our clinic if patient brings partner(s) in
- we provide HIV testing to the partner in our clinic if not known to be living with HIV
- we refer to the DOH DIS depends if the patient is known to be living with HIV – if yes, we see in our RWHAP clinic otherwise we refer out
- other, please specify: ____

24. What barriers to testing for STIs have you encountered? (select all that apply)

- supplies for STI testing are not easily accessible in exam rooms
- patient refused to provide urine for NAAT
- patient does not have insurance
- patient does not have money for insurance copay or deductible for STI testing and/or treatment
- patient refuses to have provider do NAAT swabbing (oral, anal, and/or genital)
- patient insurance does not cover lab test(s)
- patient insurance does not cover more than one screening test per year
- lack of time to conduct sexual history, STI testing, and sexual harm reduction counseling
- provider discomfort with sexual history taking and specimen collection process
- prior-authorization is needed for treatment of syphilis
- laboratory does not do oropharyngeal or rectal GC/CT NAAT testing
- insurance will not pay for oropharyngeal or rectal GC/CT NAAT testing
- insurance will not pay for oropharyngeal, genital, and rectal GC/CT NAAT testing collected at the same visit
- other, please specify: ____

25. How would you rate your clinic's lesbian, gay, bisexual, transgender, queer (LGBTQ) friendliness?
- very unfriendly
- unfriendly
- neutral
- friendly
- very friendly

26. How would you rate your clinic's lesbian, gay, bisexual, transgender, queer (LGBTQ) cultural competence?
- very LGBTQ cultural competent
- LGBTQ cultural competent
- neutral
- LGBTQ cultural incompetent
- very LGBTQ cultural incompetent

27. How would you rate your clinic's adolescent/young adult friendliness?
- very unfriendly
- unfriendly
- neutral
- friendly
- very friendly

28. How would you rate your clinic's adolescent/young adult (AYA) cultural competence?
- very AYA cultural competent
- AYA cultural competent
- neutral
- AYA cultural incompetent
- very AYA cultural incompetent

29. Which of the following is practiced or implemented in your clinic (select all that apply)?
- We have visible adolescent/young adult specific health education or awareness posters in our clinic
- We have visible gay/bisexual/MSM specific health education or awareness posters in our clinic
- We have a visible rainbow gay pride flag or a "safe space" sticker in our clinic waiting room/reception area

- We use gender non-specific terms when doing a sexual history or counseling about sexual practices
- We use terminology understood by adolescents when taking a sexual history or counseling about sexual practices
- We use the name and pronouns identified by the patient and not specifically the birth given name
- Our providers do not wear white lab coats when seeing adolescents
- We have gender neutral bathrooms in our clinic
- We tell all our adolescent patients less than 18 years old about the confidentiality law
- We interview our adolescent patients (those under 18 years) alone without parent or guardian in the room about confidential issues
- We do not use the word “homosexual” in our notes, questions, or EMR, but instead use gay, lesbian, bisexual or other terminology such as same-gendered loving, pansexual
- We allow adolescents (13-17 years) to be seen for sexuality or STI related issues in our clinic without parental permission

30. Which of the following trainings have any of the clinicians on your team had over the past year? (select all that apply and provide the clinician credentials such as MD, DO, NP, PA, or RN)

- STI testing and treatment ______________
- lesbian, gay, bisexual (LGB) or MSM health _____________________
- sexual health ________________
- taking a sexual history
- transgender care ___________________
- adolescent/young adult care _____________________
- care of pregnant individuals living with HIV ______________
- cultural sensitivity ______________________

31. Which of the following trainings have any of the non-clinical providers (case managers, patient navigators, administrators) on your team had over the past year? (select all that apply and indicate role of non-clinical provider on your team)

- STI testing and treatment ________________
- LGB or MSM health ________________
- sexual health _________________
- transgender care ________________________
- adolescent/young adult care _________________

- care of pregnant individuals living with HIV

32. Have your clinical team members participated in any of the following over the past year? (select all that apply and indicate clinician type such as MD, DO, NP, PA, RN)

- telehealth/ECHO education ________________
- state Review Boards (e.g., FIMR) __________________
- self-directed continuing education units on STIs (e.g., Grand Rounds, online modules) __________________

33. Please respond to the following questions based on your experience.

	**Always**	**Usually**	**Sometimes**	**Never**	**Unknown**
1. I'm comfortable taking a comprehensive sexual history.	☐	☐	☐	☐	☐
2. I make sure my patients completely understand me when I talk to them about their health.	☐	☐	☐	☐	☐
3. My patients accept genital or urine specimen collection for STI testing purposes.	☐	☐	☐	☐	☐
4. My patients allow me to collect anorectal specimens when needed.	☐	☐	☐	☐	☐
5. My patients can make an **acute care** appointment and be seen within 24 hours at my clinic.	☐	☐	☐	☐	☐
6. When patients contact your clinic to make a **non-acute** appointment, how often are they able to get an appointment within the next 7 days?	☐	☐	☐	☐	☐

	Always	Usually	Sometimes	Never	Unknown
7. Our clinic patients travel to the clinic from home in less than 60 minutes.	☐	☐	☐	☐	☐
8. Patient satisfaction survey results are used to improve our clinic.	☐	☐	☐	☐	☐

34. Please respond to the following questions based on your experience.

	Strongly agree	Agree	Neither agree or disagree	Disagree	Strongly Disagree
1. If a patient has gonorrhea or chlamydia in their throat or rectum, they most likely will also have it in their urine.	☐	☐	☐	☐	☐
2. Routine STI testing should be done in STD clinics or by the primary care provider, and not HIV specialists.	☐	☐	☐	☐	☐
3. Our clinic has enough evening or weekend hours for patient access.					
4. People with an STI could have avoided getting infected if they had wanted to.	☐	☐	☐	☐	☐
5. Gonorrhea, chlamydia, and syphilis are as concerning to HIV care providers as unsuppressed HIV viral loads.	☐	☐	☐	☐	☐
6. People get infected with STIs because they engage in irresponsible behaviors.	☐	☐	☐	☐	☐

THANK YOU!
