# Supplementary material for: Improving sexually transmitted infection screening, testing, and treatment among people with HIV: A mixed method needs assessment to inform a multi-site, multi-level intervention and evaluation plan
Source: PLoS One. 2021 Dec 28;16(12):e0261824. doi: 10.1371/journal.pone.0261824 (PMC8714108; doi:10.1371/journal.pone.0261824)
Supplement: S4 File — (PDF) [file pone.0261824.s004.pdf]

SEXUALLY TRANSMITTED INFECTION SCREENING READINESS CHECKLIST[1,2,3]

Administration Notes: This checklist will be administered onsite by the Rutgers University Team to the clinical team consisting of the Change Champion, a clinical prescriber (e.g., MD, DO, NP, PA) and a clinical non-prescriber (e.g., RN, SW, MA) at each of the 9 clinical demonstration sites.

Instructions: As each item is read, please indicate which response is the best fit for the given statement and most collectively represents the capacities of your clinical team, inclusive of a clinical prescriber and a clinical non-prescriber, to provide STI (i.e., chlamydia [CT], gonorrhea [GC], and syphilis) testing and treatment among people living with HIV (PLWH) or those (HIV-uninfected or unknown status) at-risk for HIV. Please allow for an estimated interview time of up to 60 minutes.

Roles of clinical demonstration site team interviewed for this checklist completion:

STAFF/CLINICAL TEAM READINESS	Yes	No	I Don't Know
**1.** Our clinic has policies and procedures in place regarding staff member(s) responsibility for prevention of HIV (for uninfected patients), CT, GC, and syphilis.			
Notes:			
**2.** Our clinic staff knows what STI screening and testing includes – including referral to other services as needed.			
Notes:			
**3.** Our clinic staff knows what STI diagnosis includes – including referral to other services as needed.			
Notes:			
**4.** Our clinic staff knows what STI treatment includes – including referral to other services as needed.			
Notes:			
**5.** There is ALL STAFF recognition of the need for STI testing, treatment, and follow-up services in our clinic.			
Notes:			

**6.** Our clinic has a way to systematically monitor STI testing, diagnosis, treatment, and follow-up data for clinic population(s).			
Notes:			
**7.** Our clinic routinely provides STI harm-reduction counseling (condom use, sex with drug use, U=U) to all patients.			
Notes:			
**8.** Our clinic has the capacity to increase CT, GC, and syphilis testing.			
Notes:			
**9.** Our clinic has the capacity to provide HIV and STI testing and treatment services of partners and people at-risk of HIV.			
Notes:			
**10.** Our clinic staff has implemented policies and procedures to allow for maximum reimbursement of STI services provided in our clinic.			
Notes:			
**11.** Our clinic has a process in use to evaluate patient care satisfaction and/or experiences regarding STI testing and treatment.			
Notes:			
**12.** Our clinic works to reduce identified barriers related to STI testing, diagnosis, treatment, and follow-up.			
Notes:			
**13.** Our clinic utilizes a range of media platforms to communicate STI information to:			
a. MSM			
b. adolescent/young adults			
c. transgender women			
d. pregnant individuals			
e. those (HIV-uninfected or unknown status) at-risk for HIV			
Notes:			
**14.** Our clinic tailors STI messages to diverse audiences including:			
a. MSM			

b. adolescent/young adults			
c. transgender women			
d. pregnant individuals			
e. those (HIV-uninfected or unknown status) at-risk for HIV			
Notes:			
**15.** Our clinic utilizes appropriate interviewing and counseling techniques for:			
a. MSM			
b. adolescent/young adults			
c. transgender women			
d. pregnant individuals			
e. those (HIV-uninfected or unknown status) at-risk for HIV			
Notes:			
**16.** Our provider(s) have time to conduct physical exams for indicators of STIs.			
Notes:			
**17.** Our provider(s) have knowledge to conduct physical exams for indicators of STIs.			
Notes:			
**18.** Our clinic has the supplies needed for GC, CT, and syphilis testing.			
Notes:			
**19.** Our clinic has the supplies needed for HIV testing.			
Notes:			
**20.** Our clinic can easily accommodate same day walk-in appointments for STI testing or treatment.			
Notes:			
**21.** Our laboratory tests extragenital site GC/CT NAAT specimens along with urine or genital site NAAT specimens.			
Notes:			

**22.** Our clinic has a policy and procedure for providing necessary follow-up care and support to patients diagnosed with an STI.			
Notes:			
**23.** Our state or local DOH provides Disease Intervention Specialist (DIS) services for GC and CT.			
Notes:			
**24.** Our state or local DOH provides DIS services for syphilis.			
Notes:			
**25.** Our state or local DOH provides DIS services for HIV.			
Notes:			

[1]Based on a template from: Centers for Disease Control and Prevention. (2005). Anti-Retroviral Treatment and Access to Services (ARTAS): An individual-level, multi-session intervention for people who are recently diagnosed with HIV: Implementation Manual. Retrieved from: www.cdc.gov/hiv/topics/cba/pdf/artas_implementation_manual.pdf

[2]AETC NCRC Mental Health Committee. Mental Health/Substance Use Care: Clinic/Health Center Readiness Assessment Tool. https://aidsetc.org/resource/mental-healthsubstance-use-care-clinichealth-center-readiness-assessment-tool

[3]Sisk K, Conneally A, Cullinen K. *Guide for Developing and Enhancing Skills in Public Health and Community Nutrition.* 3rd Ed. Public Health/Community Nutrition Practice Group of the Academy of Nutrition and Dietetics, and the Association of State Public Health Nutritionists; 2018. Available at: www.phcnpg.org. Accessed December 20, 2018.

François-Xavier Bagnoud Center, Rutgers School of Nursing
