# Supplementary material for: Improving sexually transmitted infection screening, testing, and treatment among people with HIV: A mixed method needs assessment to inform a multi-site, multi-level intervention and evaluation plan
Source: PLoS One. 2021 Dec 28;16(12):e0261824. doi: 10.1371/journal.pone.0261824 (PMC8714108; doi:10.1371/journal.pone.0261824)
Supplement: S5 File — (PDF) [file pone.0261824.s005.pdf]

CLINIC WORKFLOW OPERATIONS CHECKLIST

Administration Notes: Below is a clinic workflow checklist to be conducted by the Rutgers University team with the Change Champion in collaboration with a clinical prescriber (e.g., MD, DO, NP, PA) and a clinical non-prescriber (e.g., RN, SW, MA at each of the 9 clinical demonstration sites.

Instructions: As each question is asked, please provide a response that most collectively represents your clinical team, inclusive of a clinical prescriber and a clinical non-prescriber. Please allow for an estimated response time of up to 30 minutes.

Clinic Workflow	Yes	No	Comments
1. Patient checks in at front desk (confidential, friendly, welcoming reception).			
2. Patient waits to be seen in a waiting room.			
3. Visible in the waiting room are indications of LGBT support (rainbow flag, designated safe space sticker, images or same-sex couples on educational materials, images of transgender affirming information).			
4. Visible in the waiting room are indicators of adolescent/young adult support and friendliness (images of adolescent/young adults on pictures, pamphlets).			
5. Nurse/MA/non-clinical support staff note patient has arrived on EHR schedule.			
6. Nurse/MA/non-clinical support staff greet and/or escort patient to exam area.			
7. Nurse/MA/non-clinical support staff update patient information if needed.			
8. Nurse/MA measure and record patient’s vital signs.			

9. Nurse/MA/non-clinical support staff logs reason for visit today.			
10. Nurse/MA conducts rapid point-of-care tests (pregnancy, HIV, syphilis, GC/CT).			
11. Nurse/MA/non-clinical support staff escorts patient back to waiting room.			
12. Nurse/MA/non-clinical support staff takes patient to social worker, health educator, case manager office.			
13. Nurse/MA/non-clinical support staff takes patient to exam room to see provider.			
14. The provider greets patient, and logs onto workstation.			
15. The provider conducts a sexual history.			
16. The provider discusses HIV testing, if needed.			
17. The provider conducts a physical exam.			
18. The provider orders STI lab tests.			
19. The provider collects a genital swab chlamydia/gonorrhea NAAT.			
20. The provider collects or requests an oropharyngeal swab chlamydia/gonorrhea NAAT.			
21. The provider collects or requests a rectal swab chlamydia/gonorrhea NAAT.			

22. The provider draws blood or requests a syphilis test.			
23. The patient is asked to provide urine for chlamydia/gonorrhea NAAT.			
24. Patient self-collects swab(s) for chlamydia/gonorrhea NAAT.			
25. Patient taken to phlebotomist/lab for specimen collection.			
26. Policy exists for patient satisfaction assessment (electronic survey after visit, annual assessment done, quarterly assessment done)			

[1]Adapted from “Physician Assistant (PA) Office Visit” Health Resources and Services Administration.

François-Xavier Bagnoud Center, Rutgers School of Nursing
