# Supplementary material for: Improving sexually transmitted infection screening, testing, and treatment among people with HIV: A mixed method needs assessment to inform a multi-site, multi-level intervention and evaluation plan
Source: PLoS One. 2021 Dec 28;16(12):e0261824. doi: 10.1371/journal.pone.0261824 (PMC8714108; doi:10.1371/journal.pone.0261824)
Supplement: S6 File — (PDF) [file pone.0261824.s006.pdf]

Clinical Team Member Interview – Training

Quote	Interpretation
So we do have cultural competency training as a whole at least annually. I know recently some in-house staff did some training as far as gender sensitivity and different patients we see with like the front desk staff. Our Ryan White team participated in a culturally sensitive training done for Part A through the City maybe six months ago.	**Some training offered to staff**
I will say it's probably the same training each year, maybe a little bit of tweaks, but no, I don't think this kind of training is a—it's not like, okay, so we've learned a new thing.	**Current training does not reflect new knowledge**
We have done a little bit, but we know that's a weakness. So in our monthly office meetings, we've gone on the internet and gotten the terms, you know, just handed those out and talked about them a little bit. So we've barely just scratched the surface. I'm hoping that we can do a lot more in that area, because it's a need. It very much is a need.	**More training on cultural sensitivity**
And I'm also working now on training my resident physicians who feel awkward taking care of patients who are homosexual or transgender and they don't know how to ask these patients the right questions. So I'm working with [Name] in New Orleans where I can send out a group of my residents who are entrusted to—so that people are not judged on their sexual orientation and they feel like they can get medical care and that they're just like anybody else and then that stigma— Yeah, they're welcome. So I'm working—so those are all like some of my projects.	**Provider stigma is affecting patient care**

Clinical Team Member Interview – Clinical

Quote	Interpretation
Well I know that we do – the screenings are part of the health lab – lab draws every year for their annual, and they always have annual labs and that is part of the spring – we do the urine one, I think it is. We also do it only if they complain of symptoms or they come in and say, "Well I had a good time this weekend, so can you just check me?"	**G&C Screening is done annually or when the patient is symptomatic**
… we do a syphilis testing once a year, which is standard, I believe. But if a person feels like they have been exposed to syphilis we will test and we will order the test for them, which means that we have to contact a physician. Whether it be – where they're already here in the office or if they're doing rounds and maybe we just have to call them and be like, hey, you know, this person feels like they might be exposed to syphilis. So if they give us a verbal order then we can – then we can make a verbal order for them to go and get testing done.	**Syphilis screening is done annually or when the patient is symptomatic**
They usually are—they really don't like it. They will complain about it, but always allow us to do the swab. We always do the swab. They will complain, of course. This is just uncomfortable. I don't like to do this, but they will let us do the test.	**Patient uncomfortable with provider collection**
We had one provider who's no longer here that was doing the bulk of that, and she felt comfortable with her patients doing it because it was kind of a surprise to me. Because I was like, "Oh no, what are we doing?" And so when I spoke to him, he was like, "You know, there are times when the patient refuses and want to do it themselves." Me personally, I just I don't know if I would trust the, you know, if they're swabbing it correctly. Not saying that they can't do it, but I guess if they're instructed on how to insert it and do it, I think they are capable of doing it. But I don't think it would be a barrier.	**Provider comfortability with self-collection**

Clinical Team Member Interview – Non-clinical

Quote	Interpretation
So we do have cultural competency training as a whole at least annually. I know recently some in-house staff did some training as far as gender sensitivity and different patients we see with like the front desk staff. Our Ryan White team participated in a culturally sensitive training done for Part A through the City maybe six months ago.	**Some training offered to staff**
I will say it's probably the same training each year, maybe a little bit of tweaks, but no, I don't think this kind of training is a—it's not like, okay, so we've learned a new thing.	**Current training does not reflect new knowledge**
We have done a little bit, but we know that's a weakness. So in our monthly office meetings, we've gone on the internet and gotten the terms, you know, just handed those out and talked about them a little bit. So we've barely just scratched the surface. I'm hoping that we can do a lot more in that area, because it's a need. It very much is a need.	**More training on cultural sensitivity**
And I'm also working now on training my resident physicians who feel awkward taking care of patients who are homosexual or transgender and they don't know how to ask these patients the right questions. So I'm working with [Name] in [Louisiana] where I can send out a group of my residents who are entrusted to—so that people are not judged on their sexual orientation and they feel like they can get medical care and that they're just like anybody else and then that stigma— Yeah, they're welcome. So I'm working—so those are all like some of my projects.	**Provider stigma is affecting patient care**
