# Supplementary material for: Improving sexually transmitted infection screening, testing, and treatment among people with HIV: A mixed method needs assessment to inform a multi-site, multi-level intervention and evaluation plan
Source: PLoS One. 2021 Dec 28;16(12):e0261824. doi: 10.1371/journal.pone.0261824 (PMC8714108; doi:10.1371/journal.pone.0261824)
Supplement: S7 File — (PDF) [file pone.0261824.s007.pdf]

RESULTS OF THE PRE-INTERVENTION DATA SURVEY (2016-2017)

Sample Size (*n*) = 9

OVERALL POPULATION AND SUBPOPULATIONS FOR STUDY ENROLLMENT

CLINIC CODES
FL01
FL02
FL03
L01
L02
L03
DC01
DC02
DC03

PEOPLE LIVING WITH HIV (PLWH)

CLINIC	2016	2017
FL01	486	479
FL02	153	198
FL03	1955	1923
**FL TOTAL**	**2,594**	**2,600**
L01	376	410
L02	972	986
L03	646	611
**LA TOTAL**	**1,994**	**2,007**
DC01	85	85
DC02	559	510
DC03	111	136
**DC TOTAL**	**755**	**731**
**OVERALL TOTAL**	**5343**	**5338**

INDIVIDUALS (HIV-UNINFECTED OR UNKNOWN HIV STATUS) AT-RISK FOR HIV INFECTION

CLINIC	2016	2017
FL01	0	0
FL02	0	0
FL03	0	0
**FL TOTAL**	**0**	**0**
L01	1200	1500
L02	0	0
L03	34	89
**LA TOTAL**	**1,234**	**1,589**
DC01	90	90
DC02	0	0
DC03	0	0
**DC TOTAL**	**90**	**90**
**OVERALL TOTAL**	**1324**	**1679**

ADOLESCENTS/YOUNG ADULTS

CLINIC	2016	2017
FL01	0	0
FL02	31	34
FL03	23	94
**FL TOTAL**	**54**	**128**
L01	14	25
L02	144	180
L03	99	82
**LA TOTAL**	**257**	**287**
DC01	20	23
DC02	0	0
DC03	35	47
**DC TOTAL**	**55**	**70**
**OVERALL TOTAL**	**366**	**485**

TRANSGENDER WOMEN LIVING WITH HIV

CLINIC	2016	2017
FL01	0	0
FL02	0	0
FL03	0	31
**FL TOTAL**	**0**	**31**
L01	0	2
L02	0	0
L03	4	4
**LA TOTAL**	**4**	**6**
DC01	3	2
DC02	0	0
DC03	2	2
**DC TOTAL**	**5**	**4**
**OVERALL TOTAL**	**9**	**41**

PREGNANT INDIVIDUALS LIVING WITH HIV

CLINIC	2016	2017
FL01	2	4
FL02	0	0
FL03	9	54
**FL TOTAL**	**11**	**58**
L01	0	1
L02	64	62
L03	8	8
**LA TOTAL**	**72**	**71**
DC01	0	0
DC02	0	0
DC03	2	2
**DC TOTAL**	**2**	**2**
**OVERALL TOTAL**	**85**	**131**

MEN WHO HAVE SEX WITH MEN (MSM) LIVING WITH HIV

CLINIC	2016	2017
FL01	0	0
FL02	0	0
FL03	85	757
**FL TOTAL**	**85**	**757**
L01	98	135
L02	0	0
L03	136	142
**LA TOTAL**	**234**	**277**
DC01	0	0
DC02	0	0
DC03	72	85
**DC TOTAL**	**72**	**85**
**OVERALL TOTAL**	**391**	**1119**
