# Supplementary material for: Improving sexually transmitted infection screening, testing, and treatment among people with HIV: A mixed method needs assessment to inform a multi-site, multi-level intervention and evaluation plan
Source: PLoS One. 2021 Dec 28;16(12):e0261824. doi: 10.1371/journal.pone.0261824 (PMC8714108; doi:10.1371/journal.pone.0261824)
Supplement: S8 File — (PDF) [file pone.0261824.s008.pdf]

RESULTS OF THE CLINICAL TEAM MEMBER PROCESS, ATTITUDES, & BELIEFS SURVEY

Sample Size (*n*) = 27: Change Champions, Clinical Prescribers, and Clinical non-Prescribers

KEY AGGREGATE FINDINGS

Sexual History Taking

- 44% conduct a consistent, comprehensive sexual history on intake
- 74% conduct follow-up sexual histories at acute care visits when symptomatic for an STI

STI Testing

- Among sexually active adolescents and adults living with HIV
  - 67% test for STIs on at least an annual basis
  - 18% test for STIs every 3-4 months
  - 78% test for STIs if symptomatic for an STI
- 59% of patients self-collect NAAT or culture specimens for GC and CT

STI Treatment

- 52% and 48% of patients are brought back into clinic for a positive STI test result after being tested within 1-3 days and 4-10 days, respectively.

Clinical Barriers to STI Testing and Treatment

- The top four intervenable barriers include the following:
  - Patient refuses to have provider do NAAT swabbing (oral, anal, and/or genital)
  - Time needed for testing, and sexual harm reduction counseling
  - Provider discomfort with sexual history taking and specimen collection process
  - Supplies for STI testing are not easily accessible in exam rooms

Non-Clinical Barriers to STI Testing and Treatment

- 25% and 26% of respondents rated their clinics as less than friendly to LGBTQ individuals and adolescent/young adults, respectively.
- 37% of respondents rated their clinics as less than culturally competent for both LGBTQ individuals and adolescent/young adults.
- More than 50% of respondents reported that their clinics **do not**:
  - have visible adolescent/young adult specific health education or awareness posters;
  - have a visible rainbow gay pride flag or a "safe space" sticker in the clinical waiting room/reception area;
  - use the words gay, lesbian, bisexual or other terminology such as same-gendered loving, pansexual, but instead use "homosexual" in notes, questions, or EMR;
  - use gender non-specific terms when doing a sexual history or counseling about sexual practices;
  - have gender neutral bathrooms;
  - allow adolescents (13-17 years) to be seen for sexuality or STI related issues without parental/guardian permission;
  - interview adolescent patients (those under 18 years) about confidential issues without a parent or guardian in the room;

- tell all adolescent patients less than 18 years old about the confidentiality law; and
- have providers that do not wear white lab coats when seeing adolescents

Clinical Provider Training

- Provider training opportunities exist across clinical disciplines with 26-56% of respondents reporting a lack of training at their clinics over the past year on the following topics:
  - STI Testing and Treatment
  - LGB or MSM Health
  - Sexual Health
  - Sexual History
  - Transgender Care
  - Adolescent/Young Adult Care
  - Caring for Pregnant Individuals Living with HIV
  - Cultural Sensitivity

Non-Clinical Provider Training

- Non-clinical provider training opportunities exist among non-clinical staff with 44-67% of respondents reporting a lack of training at their clinics over the past year on the following topics:
  - STI Testing and Treatment
  - LGB or MSM Health
  - Sexual Health
  - Transgender Care
  - Adolescent/Young Adult Care
  - Caring for Pregnant Individuals Living with HIV

Clinical and Non-Clinical Provider Attitudes and Beliefs

- 29% of respondents reported that if a patient has gonorrhea or chlamydia in their throat or rectum, they most likely will also have it in their urine.
- 29% of respondents reported that routine STI testing should be done in STD clinics or by the primary care provider, and not HIV specialists.
- 48% of respondents reported that their clinics do not have enough evening or weekend hours for patient access.
- 26% of respondents reported that people with an STI could have avoided getting infected if they had wanted to.

CLINICAL

Current Processes: Sexual History Taking

Sexual History Taking
(select all that apply)

Mode	Percent (%)
Paper OR online survey prior to coming to clinic	0
Patient completed online survey in clinic prior to being seen by provider(s), such as computer-assisted self-interview or CASI	0
Prescribing provider asks patient questions	85
Non-prescribing provider asks patient questions	59
Paper survey in clinic prior to being seen by provider(s)	15

Current Processes: STI Testing

Tests Done at Intake Based on Sexual History
(select all that apply)

ADOLESCENTS AND ADULTS LIVING WITH HIV Percent (%)				
**TEST**	**Women**	**Men**	**Transgender Women**	**Transgender Men**
Syphilis RPR/FTA	96	96	96	93
Syphilis IgG	22	22	22	22
Urine GC/CT NAAT	85	93	93	89
None	4	4	4	7

Tests Done at Intake Based on Sexual History
(select all that apply)

ADOLESCENTS AND ADULTS HIV-UNINFECTED BUT AT RISK FOR INFECTION	
**TEST**	**Percent (%)**
Syphilis RPR/FTA	82
Syphilis IgG	18
Urine GC/CT NAAT	78
Rapid HIV antigen/antibody	48
Non-rapid HIV antigen/antibody	44
None	4
STI testing not provided to HIV-uninfected or status unknown people	15

If patient wants to be seen for a possible STI (testing and/or treatment) and does not have an appointment with your clinic, what would be your clinic's most likely response?

RESPONSE	Percent (%)
Come into the clinic and we'll make sure you get seen today	78
Let's make you an appointment to come in to the clinic, but it will not be today	15
We're going to have to have you go to the DOH STD clinic or another provider to be seen	4
You should go to the emergency department or urgent care center	4

Who collects the NAAT or culture specimens for GC and CT in your clinic? (select all that apply)

Specimen Collection	Percent (%)
Patient self-collects	59
Prescribing provider collects	52
Non-prescribing clinical team member collects	44
We do not do GC/CT NAAT or GC cultures in our HIV care clinic	4

Current Processes: STI Treatment

If an STI test result comes back positive for an asymptomatic patient who is tested, who notifies the patient to come back to the clinic for treatment or to take medication(s) called into the patient's pharmacy? (select all that apply)

Clinical Team Member	Percent (%)
Prescribing Provider	59
Nurse	74
Case Manager	7
Patient Navigator	4
Social Worker	4
Other (e.g., DIS, MA)	30

On the average, how quickly are patients brought back into clinic for a positive STI test result after being tested?

Timeframe	Percent (%)
Within 1-3 days	52
Within 4-6 days	33
Within 7-10 days	15

Barriers to Testing and Treatment

What barriers to testing and treatment of STIs have you encountered? (select all that apply)

Barriers	Percent (%)
Patient refuses to have provider do NAAT swabbing (oral, anal, and/or genital)	44
Patient does not have insurance	33
Patient does not have money for insurance copay or deductible for STI testing and/or treatment	33
Time needed for testing, and sexual harm reduction counseling	33
Other (e.g., client does not show for appointment or treatment)	30
Patient refuses to provide urine for NAAT	26
Patient insurance does not cover more than one screening test per year	18
Provider discomfort with sexual history taking and specimen collection process	18
Patient insurance does not cover lab test(s)	15
Supplies for STI testing are not easily accessible in exam rooms	15
Laboratory does not do oropharyngeal or rectal GC/CT NAAT testing	15
Insurance will not pay for oropharyngeal or rectal GC/CT NAAT testing	7
Insurance will not pay for oropharyngeal, genital, and rectal GC/CT NAAT testing collected at the same visit	4
Prior-authorization is needed for treatment of syphilis	0

NON-CLINICAL

How would you rate your clinic's friendliness?

Friendliness	Percent (%)	
	LGBTQ	Adolescent/Young Adult
Very unfriendly	7	4
Unfriendly	1	4
Neutral	18	18
Friendly	41	56
Very friendly	33	18

How would you rate your clinic's cultural competence?

Cultural Competence	Percent (%)	
	LGBTQ	Adolescent/Young Adult
Very cultural competent	33	22
Cultural competent	30	41
Neutral	33	26
Cultural incompetent	4	7
Very cultural incompetent	0	4

Which of the following is practiced or implemented in your clinic? (select all that apply)

Practiced or Implemented	Percent (%)
Visible gay/bisexual/MSM specific health education or awareness posters	67
Use name and pronouns identified by the patient and not specifically the birth given name	67
Use terminology understood by adolescents when taking a sexual history or counseling about sexual practices	56
Visible adolescent/young adult specific health education or awareness posters	48
Do not use the word "homosexual" in our notes, questions, or EMR, but instead use gay, lesbian, bisexual or other terminology such as same-gendered loving, pansexual	44
Use gender non-specific terms when doing a sexual history or counseling about sexual practices	41
Gender neutral bathrooms	33
Allow adolescents (13-17 years) to be seen for sexuality or STI related issues without parental/guardian permission	33
Interview our adolescent patients (those under 18 years) alone without parent or guardian in the room about confidential issues	30
Tell all our adolescent patients less than 18 years old about the confidentiality law	26
Visible rainbow gay pride flag or a "safe space" sticker in our waiting room/reception area	15
Providers do not wear white lab coats when seeing adolescents	15

PROVIDER TRAINING

Over the past year, which of the following types of CLINICIANS on your team attended training(s) on

**Training Topic**	**Percent (%)**					
	MD	DO	NP	PA	RN	N/A
STI Testing and Treatment	67	4	56	7	30	26
LGB or MSM Health	52	4	44	7	26	41
Sexual Health	63	4	56	11	37	30
Sexual History	44	0	41	7	15	48
Transgender Care	48	0	37	7	18	41
Adolescent/Young Adult Care	41	0	30	4	18	56
Caring for Pregnant Individuals Living with HIV	41	4	44	4	18	41
Cultural Sensitivity	63	0	48	7	30	30
Telehealth/ECHO Education	30	4	18	7	7	70
Meeting(s): State Review Board (e.g., FIMR)	15	0	4	0	4	85
Self-Directed Continuing Education Units on STIs (e.g., Grand Rounds, online modules)	52	0	48	4	22	37

NON-CLINICAL PROVIDER TRAINING

Over the past year, which of the following types of NON-CLINICAL PROVIDERS on your team attended training(s) on

Training Topic	Percent (%)			
	Case Managers	Patient Navigators	Administrators	N/A
STI Testing and Treatment	48	44	18	44
LGB or MSM Health	41	37	30	44
Sexual Health	41	33	18	56
Transgender Care	41	37	26	44
Adolescent/Young Adult Care	26	26	11	67
Caring for Pregnant Individuals Living with HIV	30	18	11	67

Clinical and Non-Clinical Provider Attitudes and Beliefs

Please rate the following statements based on your experience.

Statement	Percent (%)				
	Always	Usually	Sometimes	Never	Unknown
I'm comfortable taking a comprehensive sexual history.	63	15	7	0	15
I make sure my patients completely understand me when I talk to them about their health.	59	26	0	0	15
My patients accept genital or urine specimen collection for STI testing purposes.	44	37	4	0	15
My patients allow me to collect anorectal specimens when needed.	18	37	4	4	37
My patients can make an acute care appointment and be seen within 24 hours at my clinic.	30	33	26	4	7
When patients contact our clinic to make a non-acute appointment, they can get an appointment within the next 7 days.	33	41	18	4	4
Our patients travel to the clinic from home in less than 60 minutes.	15	59	11	0	15
Patient satisfaction survey results are used to improve our clinic.	37	37	11	0	15

Please rate the following statements based on your experience.

Statement	Percent (%)				
	Strongly Agree	Agree	Neither Agree or Disagree	Disagree	Strongly Disagree
If a patient has gonorrhea or chlamydia in their throat or rectum, they most likely will also have it in their urine.	18	11	30	37	4
Routine STI testing should be done in STD clinics or by the primary care provider, and not HIV specialists.	22	7	7	33	30
Our clinic has enough evening or weekend hours for patient access.	0	22	30	30	18
People with an STI could have avoided getting infected if they had wanted to.	0	26	30	37	7
Gonorrhea, chlamydia, and syphilis are as concerning to HIV care providers as unsuppressed HIV viral loads.	26	37	15	18	4
People get infected with STIs because they engage in irresponsible behaviors.	11	11	33	44	0
