# Supplementary material for: Improving sexually transmitted infection screening, testing, and treatment among people with HIV: A mixed method needs assessment to inform a multi-site, multi-level intervention and evaluation plan
Source: PLoS One. 2021 Dec 28;16(12):e0261824. doi: 10.1371/journal.pone.0261824 (PMC8714108; doi:10.1371/journal.pone.0261824)
Supplement: S9 File — (PDF) [file pone.0261824.s009.pdf]

RESULTS OF THE STI SCREENING READINESS CHECKLIST

Sample Size (*n*) = 9: Clinical Team (Change Champion, Clinical Prescriber (e.g., MD, DO, NP, PA), and Clinical non-Prescriber (e.g., RN, SW, MA) at each of the nine clinical demonstration sites

KEY AGGREGATE FINDINGS

STI Prevention, Screening, Testing, Diagnosis, and Treatment

100% of clinics report

- staff knowledge of STI screening, testing, diagnosis, and treatment;
- clinic capacity to increase CT, GC, and syphilis testing;
- provider time to conduct physical exams for indicators of STIs;
- provider knowledge to conduct physical exams for indicators of STIs;
- having the supplies needed for GC, CT, and syphilis testing; and
- working to reduce identified barriers related to STI testing, diagnosis, treatment, and follow-up

89% of clinics report

- laboratory testing of extragenital site GC/CT NAAT specimens along with urine or genital site NAAT specimens; and
- a policy and procedure for providing necessary follow-up care and support to patients diagnosed with an STI

78% of clinics report

- having a way to systematically monitor STI testing, diagnosis, treatment, and follow-up data for clinic population(s); and
- routine provision of STI harm-reduction counseling (condom use, sex with drug use, U=U) to all patients

- 67% of clinics report having the capacity to provide HIV and STI testing and treatment services of partners and people at-risk of HIV

44% of clinics report

- implementation of policies and procedures by clinic staff to allow for maximum reimbursement of STI services provided; and
- a process in use to evaluate patient care satisfaction and/or experiences regarding STI testing and treatment

- 33% of clinics report having policies and procedures in place regarding staff member(s) responsibility for prevention of HIV (for uninfected patients), CT, GC, and syphilis

Clinical Barriers to and Supports for STI Testing and Treatment

100% of clinics report

- state or local DOH provision of DIS services for syphilis; and
- state or local DOH provision of DIS services for HIV

- 33% of clinics report state or local DOH provision of DIS services for GC and CT

- 89% of clinics report they can easily accommodate same day walk-in appointments for STI testing or treatment

- 67% of clinics report they have the supplies needed for HIV testing

Non-Clinical Barriers to and Support for STI Testing and Treatment

44-67% of clinics report using a range of media platforms to communicate STI information to

- MSM (67%)
- pregnant individuals (56%)
- adolescent/young adults (44%)
- those (HIV-uninfected or unknown status) at-risk for HIV (44%)
- transgender women (11%)

33-56% of clinics report tailoring STI messages to diverse audiences including

- pregnant individuals (56%)
- transgender women (56%)
- MSM (44%)
- adolescent/young adults (44%)
- those (HIV-uninfected or unknown status) at-risk for HIV (33%)

Clinical Provider Training

56-78% of clinics report utilizing appropriate interviewing and counseling techniques for

- MSM (78%)
- pregnant individuals (78%)
- transgender women (67%)
- adolescent/young adults (67%)
- those (HIV-uninfected or unknown status) at-risk for HIV (56%)

SURVEY #3 - SCREENING READINESS CHECKLIST[1,2,3]

Sample Size (*n*) = 9: Clinical Team (Change Champion, Clinical Prescriber (e.g., MD, DO, NP, PA), and Clinical non-Prescriber (e.g., RN, SW, MA) at each of the nine clinical demonstration sites

STAFF/CLINICAL TEAM READINESS	Percent (%)		
	Yes	No	I Don't Know
**1.** Our clinic has policies and procedures in place regarding staff member(s) responsibility for prevention of HIV (for uninfected patients), CT, GC, and syphilis.	33	56	11
Notes:			
**2.** Our clinic staff knows what STI screening and testing includes – including referral to other services as needed.	100	0	0
Notes:			
**3.** Our clinic staff knows what STI diagnosis includes – including referral to other services as needed.	100	0	0
Notes:			
**4.** Our clinic staff knows what STI treatment includes – including referral to other services as needed.	100	0	0
Notes:			
**5.** There is ALL STAFF recognition of the need for STI testing, treatment, and follow-up services in our clinic.	89	11	0
Notes:			
**6.** Our clinic has a way to systematically monitor STI testing, diagnosis, treatment, and follow-up data for clinic population(s).	78	22	0
Notes:			
**7.** Our clinic routinely provides STI harm-reduction counseling (condom use, sex with drug use, U=U) to all patients.	78	22	0
Notes:			
**8.** Our clinic has the capacity to increase CT, GC, and syphilis testing.	100	0	0
Notes:			
**9.** Our clinic has the capacity to provide HIV and STI testing and treatment services of partners and people at-risk of HIV.	67	33	0

Notes:			
**10.** Our clinic staff has implemented policies and procedures to allow for maximum reimbursement of STI services provided in our clinic.	44	33	22
Notes:			
**11.** Our clinic has a process in use to evaluate patient care satisfaction and/or experiences regarding STI testing and treatment.	44	56	0
Notes:			
**12.** Our clinic works to reduce identified barriers related to STI testing, diagnosis, treatment, and follow-up.	100	0	0
Notes:			
**13.** Our clinic utilizes a range of media platforms to communicate STI information to:			
a. MSM	67	33	0
b. adolescent/young adults	44	44	11
c. transgender women	11	78	11
d. pregnant individuals	56	44	0
e. those (HIV-uninfected or unknown status) at-risk for HIV	44	22	33
Notes:			
**14.** Our clinic tailors STI messages to diverse audiences including:			
a. MSM	44	33	22
b. adolescent/young adults	44	44	11
c. transgender women	56	33	11
d. pregnant individuals	56	33	11
e. those (HIV-uninfected or unknown status) at-risk for HIV	33	11	56
Notes:			
**15.** Our clinic utilizes appropriate interviewing and counseling techniques for:			
a. MSM	78	22	0
b. adolescent/young adults	67	33	0
c. transgender women	67	33	0
d. pregnant individuals	78	22	0
e. those (HIV-uninfected or unknown status) at-risk for HIV	56	11	33
Notes:			
**16.** Our provider(s) have time to conduct physical exams for indicators of STIs.	100	0	0
Notes:			

**17.** Our provider(s) have knowledge to conduct physical exams for indicators of STIs.	100	0	0
Notes:			
**18.** Our clinic has the supplies needed for GC, CT, and syphilis testing.	100	0	0
Notes:			
**19.** Our clinic has the supplies needed for HIV testing.	67	11	22
Notes:			
**20.** Our clinic can easily accommodate same day walk-in appointments for STI testing or treatment.	89	11	0
Notes:			
**21.** Our laboratory tests extragenital site GC/CT NAAT specimens along with urine or genital site NAAT specimens.	89	11	0
Notes:			
**22.** Our clinic has a policy and procedure for providing necessary follow-up care and support to patients diagnosed with an STI.	89	11	0
Notes:			
**23.** Our state or local DOH provides Disease Intervention Specialist (DIS) services for GC and CT.	33	67	0
Notes:			
**24.** Our state or local DOH provides DIS services for syphilis.	100	0	0
Notes:			
**25.** Our state or local DOH provides DIS services for HIV.	100	0	0
Notes:			

[1]Based on a template from: Centers for Disease Control and Prevention. (2005). Anti-Retroviral Treatment and Access to Services (ARTAS): An individual-level, multi-session intervention for people who are recently diagnosed with HIV: Implementation Manual. Retrieved from: www.cdc.gov/hiv/topics/cba/pdf/artas_implementation_manual.pdf

[2]AETC NCRC Mental Health Committee. Mental Health/Substance Use Care: Clinic/Health Center Readiness Assessment Tool. https://aidsetc.org/resource/mental-healthsubstance-use-care-clinichealth-center-readiness-assessment-tool

[3]Sisk K, Conneally A, Cullinen K. *Guide for Developing and Enhancing Skills in Public Health and Community Nutrition.* 3rd Ed. Public Health/Community Nutrition Practice Group of the Academy of Nutrition and Dietetics, and the Association of State Public Health Nutritionists; 2018. Available at: www.phcnpg.org. Accessed December 20, 2018.
