# Supplementary material for: Improving sexually transmitted infection screening, testing, and treatment among people with HIV: A mixed method needs assessment to inform a multi-site, multi-level intervention and evaluation plan
Source: PLoS One. 2021 Dec 28;16(12):e0261824. doi: 10.1371/journal.pone.0261824 (PMC8714108; doi:10.1371/journal.pone.0261824)
Supplement: S10 File — (PDF) [file pone.0261824.s010.pdf]

RESULTS OF THE CLINIC WORKFLOW OPERATIONS CHECKLIST

Sample Size (*n*) = 9: Clinical Team (Change Champion, Clinical Prescriber (e.g., MD, DO, NP, PA), and Clinical non-Prescriber (e.g., RN, SW, MA) at each of the nine clinical demonstration sites

KEY AGGREGATE FINDINGS

Routine clinic workflow operations were observed at each of the nine clinical demonstration sites.

Most noteworthy observations are summarized below.

STI Prevention, Screening, Testing, Diagnosis, and Treatment

- 100% of providers conduct a sexual history

- 100% of patients are asked to provide urine for chlamydia/gonorrhea NAAT

- 67% of patients self-collect swab(s) for chlamydia/gonorrhea NAAT

89% of providers

  - collect or request an oropharyngeal swab chlamydia/gonorrhea NAAT; and
  - collect or request a rectal swab chlamydia/gonorrhea NAAT

- 56% of providers collect a genital swab chlamydia/gonorrhea NAAT

- 78% of providers discuss HIV testing, if needed

- 78% of Nurses/MAs conduct rapid point-of-care tests (pregnancy, HIV, syphilis, GC/CT)

- 89% of clinics have a policy for patient satisfaction assessment (electronic survey after visit, annual assessment done, quarterly assessment done)

Non-Clinical Barriers to and Support for STI Testing and Treatment

- 44% of clinic waiting rooms have visible indications of LGBT support (rainbow flag, designated safe space sticker, images or same-sex couples on educational materials, images of transgender affirming information)

- 56% of clinic waiting rooms have visible indicators of adolescent/young adult support and friendliness (images of adolescent/young adults on pictures, pamphlets)

CLINIC WORKFLOW OPERATIONS CHECKLIST

Sample Size (*n*) = 9: Clinical Team (Change Champion, Clinical Prescriber (e.g., MD, DO, NP, PA), and Clinical non-Prescriber (e.g., RN, SW, MA) at each of the nine clinical demonstration sites

Clinic Workflow	Percent (%)		Comments
	Yes	No	
**1.** Patient checks in at front desk (confidential, friendly, welcoming reception).	100	0	
**2.** Patient waits to be seen in a waiting room.	100	0	
**3.** Visible in the waiting room are indications of LGBT support (rainbow flag, designated safe space sticker, images or same-sex couples on educational materials, images of transgender affirming information).	44	56	
**4.** Visible in the waiting room are indicators of adolescent/young adult support and friendliness (images of adolescent/young adults on pictures, pamphlets).	56	44	
**5.** Nurse/MA/non-clinical support staff note patient has arrived on EHR schedule.	100	0	
**6.** Nurse/MA/non-clinical support staff greet and/or escort patient to exam area.	100	0	
**7.** Nurse/MA/non-clinical support staff update patient information if needed.	100	0	

**8.** Nurse/MA measure and record patient's vital signs.	100	0	
**9.** Nurse/MA/non-clinical support staff logs reason for visit today.	100	0	
**10.** Nurse/MA conducts rapid point-of-care tests (pregnancy, HIV, syphilis, GC/CT).	78	22	
**11.** Nurse/MA/non-clinical support staff escorts patient back to waiting room.	56	33	
**12.** Nurse/MA/non-clinical support staff takes patient to social worker, health educator, case manager office.	67	33	
**13.** Nurse/MA/non-clinical support staff takes patient to exam room to see provider.	89	11	
**14.** The provider greets patient, and logs onto workstation.	100	0	
**15.** The provider conducts a sexual history.	100	0	
**16.** The provider discusses HIV testing, if needed.	78	22	
**17.** The provider conducts a physical exam.	100	0	
**18.** The provider orders STI lab tests.	100	0	

**19.** The provider collects a genital swab chlamydia/gonorrhea NAAT.	56	44	
**20.** The provider collects or requests an oropharyngeal swab chlamydia/gonorrhea NAAT.	89	11	
**21.** The provider collects or requests a rectal swab chlamydia/gonorrhea NAAT.	89	11	
**22.** The provider draws blood or requests a syphilis test.	89	11	
**23.** The patient is asked to provide urine for chlamydia/gonorrhea NAAT.	100	0	
**24.** Patient self-collects swab(s) for chlamydia/gonorrhea NAAT.	67	33	
**25.** Patient taken to phlebotomist/lab for specimen collection.	100	0	
**26.** Policy exists for patient satisfaction assessment (electronic survey after visit, annual assessment done, quarterly assessment done)	89	11	

[1]Adapted from "Physician Assistant (PA) Office Visit" Health Resources and Services Administration.
